# Bridging research and clinical care: A clinic‐based registry and biorepository for accelerating Alzheimer's diagnostics

**DOI:** 10.1002/alz70858_106662

**Published:** 2025-12-24

**Authors:** Jeffrey M. Burns, Eric D Vidoni, Casey S. John, Hana Mayfield, Dinesh Pal Mudaranthakam, Sam Pepper, Rebecca Bothwell, Melissa Dodd, Becca Johnson, Rebecca J Lepping, Robyn A Honea, Jill K Morris

**Affiliations:** ^1^ University of Kansas Alzheimer's Disease Research Center, Kansas City, KS, USA; ^2^ University of Kansas Medical Center, Alzheimer's Disease Research Center, Fairway, KS, USA; ^3^ University of Kansas Alzheimer's Disease Research Center, Fairway, KS, USA; ^4^ University of Kansas Medical Center, Kansas City, KS, USA; ^5^ University of Kansas Alzheimer's Disease Center, Fairway, KS, USA; ^6^ University Of Kansas Alzheimer's Disease Research Center, Fairway, KS, USA

## Abstract

**Background:**

Integrating research into clinical care presents an opportunity to accelerate the diagnosis and treatment of ADRD. We piloted the Brain Health Care Accelerator (BHCA) Registry and Biorepository to transform the Memory Care Clinic into a research engine by embedding informed consent, clinical data collection, imaging, and biosample acquisition into routine patient care for research purposes. A key objective is to integrate the data into the Global Research and Imaging Platform (GRIP), enabling researchers to access and interact with the data.

**Method:**

KU Memory Clinic patients enroll in the BHCA through an electronic informed consent that allows for the collection and sharing of EMR data, cognitive testing results, neuroimaging, and biosamples (blood and CSF) from standard‐of‐care procedures. Consent is signed in clinic or at home with no additional research‐related procedures. Participants also consent to the sharing of their data and samples with approved research projects under a governance protocol. As a use case, the BHCA registry provided biosamples to the ADRC Biomarker Core for an IRB‐approved validation study of pTau217 (Lumipulse), supporting broader biomarker validation efforts.

**Result:**

Between June 2023 and January 2025, *n* = 269 patients were enrolled in the BHCA Registry (mean age = 74.1(7.9), 51.7% female, 94.1% White, 93.7% Not Hispanic or Latino), including 10 cognitively normal individuals, 116 with mild cognitive impairment (MCI), 96 with dementia/AD, and 47 with another cognitive diagnosis. A total of 158 plasma and 75 CSF samples have been banked for future sharing. We are currently developing a system to identify and catalog available brain MRI and amyloid PET scans from these patients, enabling efficient retrieval and analysis for future research. To date we have shared a total of 62 plasma samples with the biomarker core to assist in the validation of our plasma pTau217.

**Conclusion:**

The BHCA successfully bridges research and clinical care, demonstrating the feasibility of embedding biosample and real‐world data collection into clinical workflows through a low‐cost, scalable model. We plan to expand enrollment to other clinical sites and, deploy this dataset in the GRIP environment (in partnership with Gates Ventures) providing an interactive dashboard for researchers to accelerate discoveries using real‐world clinical data

